# Morphological instability and cancer invasion: a 'splashing water drop' analogy

**DOI:** 10.1186/1742-4682-4-4

**Published:** 2007-01-25

**Authors:** Caterina Guiot, Pier P Delsanto, Thomas S Deisboeck

**Affiliations:** 1 Dip. Neuroscience and CNISM, Università di Torino, Italy; 2 Dip. Fisica, Politecnico di Torino, Italy; 3 Complex Biosystems Modeling Laboratory, Harvard-MIT (HST) Athinoula A. Martinos Center for Biomedical Imaging, Massachusetts General Hospital, Charlestown, MA 02129, USA

## Abstract

**Background:**

Tissue invasion, one of the hallmarks of cancer, is a major clinical problem. Recent studies suggest that the process of invasion is driven at least in part by a set of physical forces that may be susceptible to mathematical modelling which could have practical clinical value.

**Model and conclusion:**

We present an analogy between two unrelated instabilities. One is caused by the impact of a drop of water on a solid surface while the other concerns a tumor that develops invasive cellular branches into the surrounding host tissue. In spite of the apparent abstractness of the idea, it yields a very practical result, i.e. an index that predicts tumor invasion based on a few measurable parameters. We discuss its application in the context of experimental data and suggest potential clinical implications.

## Background

Tissue invasion is one of the hallmarks of cancer [1]. From the primary tumor mass, cells are able to move out and infiltrate adjacent tissues by means of degrading enzymes (e.g., [2]). Depending on the cancer type, these cells may form distant settlements, i.e. metastases (e.g., [3]). Tumor expansion therefore results from the complex interplay between the developmental ability of the tumor itself and the characteristics of the host tissue in which its growth occurs (e.g., [4]).

**Figure 1:**
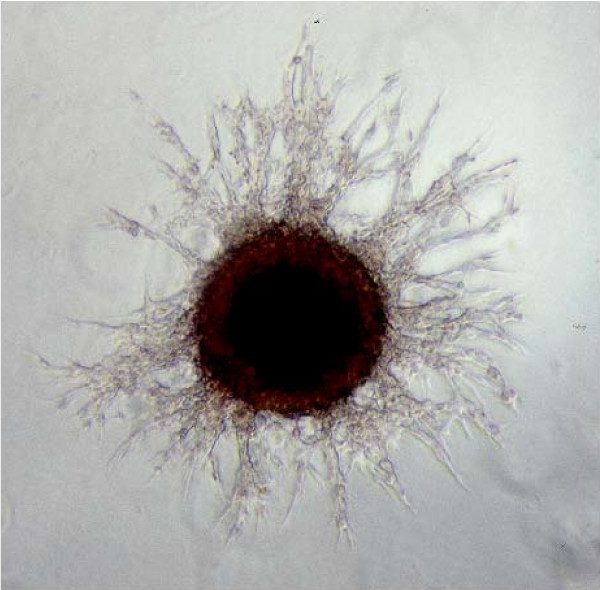
Microscopy image of a multicellular tumor spheroid, exhibiting an extensive branching system that rapidly expands into the surrounding extracellular matrix gel. These branches consist of multiple invasive cells. (Reprinted from Habib et al. [33], with permission from Elsevier).

It has been recently proposed [5] that cancer invasion can be described as a morphological *instability *that occurs during solid tumor growth and results in invasive 'fingering', i.e. branching patterns (see Figure 1). This instability may be driven by any physical or chemical condition (oxygen, glucose, acid and drug concentration gradients), provided that the average cohesion among tumor cells decreases and/or their adhesion to the stroma increases (for a recent review on related molecular aspects, such as the cadherin-'switch', see [6]). In fact, the aforementioned model of Cristini et al. [5] shows that reductions in the *surface tension *at the tumor-tissue interface may generate and control tumor branching in the nearby tissues. A previous investigation from the same group [7] had analyzed different tumor growth regimes and shown that invasive fingering *in vivo *could be driven by vascular and elastic anisotropies in highly vascularized tumors. A recent advance [8] shows that the competition between proliferation (shape-destabilizing force) and adhesion (shape-stabilizing force) can be implemented in a more general mathematical description of tumor growth and accounts for many experimental evidences. Correspondingly, another recent paper by Anderson [9] stresses the relevance of cell adhesion in the process of tumor invasion.

**Figure 2:**
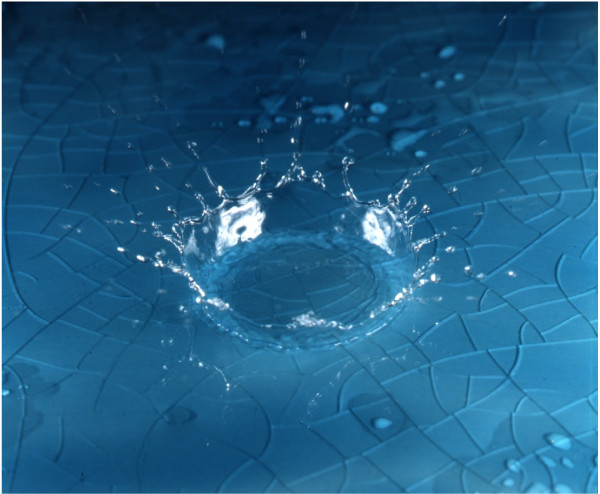
Water drop impact on a solid surface. (Courtesy Adam Hart-Davis/DHD Multimedia Gallery [34]).

Undoubtedly, a more detailed insight into the mechanisms that drive tumor invasion is critical for targeting these cancer cell populations more effectively, and, possibly, concepts derived from other scientific disciplines may contribute valuable insights. It is in this line of thought that we propose an *analogy *with the case of a liquid drop, which impacts on a solid surface and causes the formation of a fluid 'crown'. Such instability, termed Rayleigh or Yarin-Weiss capillary instability, has been extensively studied in the field of fluid dynamics [10-12] (see Figure 2).

Both phenomena share similar features, such as secondary jets (corresponding to the invasive branching in the case of tumors), nucleation near the fluid rim (corresponding to the evidence for branching confluence) and dispersion of small drops at the fluid-air interface (with resemblance to proliferating aggregates that have been reported to emerge within the invasive cell population [13].

## The Model

Fluid dynamicists describe their system by means of some non-dimensional numbers, such as the Weber number We = ρD V^2^/σ, the Ohnesorge number Oh = μ/sqrt(ρσD), the Reynold number Re = ρDV/μ and the Capillary number Ca = μV/σ, where ρ is the fluid density, D the drop diameter, V the impact velocity, μ the fluid viscosity and σ the surface tension. For instance, [14] showed that the splash/non-splash boundary for several different fluids is well described by sqrt(Ca) = 0.35. Provided an estimate for both tumor viscosity and surface tension is available, it would be interesting to investigate whether similar non-dimensional quantities could discriminate between invasive and non invasive tumor behaviour. Moreover, also the number of invasive branches may be predicted on the basis of the previous nondimensional numbers [15]. According to these authors the number of branches is given by:

N_f _= 2πR/λ     (1)

λ = 2π(3σ/ρa)^1/2 ^    (2)

where R is the radius of the drop and *a *is the 'deceleration' at the impact. In the case of a splashing drop, the impact velocity is the main measurable parameter related to the way in which kinetic energy is converted into surface energy, associated with the increased free-surface area and viscous dissipation. Splashing is in some sense the droplet's reaction to a sudden increase in pressure. The relevant quantity, due to the very short time scale involved in the process, is the splashing impact.

In the case of an invading tumor, however, the concept of 'impact' cannot be used; the increased mechanical pressure, exerted by the confining microenvironment due to cancer expansion, elicits a much slower response, hence the instability develops over a much larger time scale. This suggests that, equivalently, the tumor cell-matrix interaction could be a critical parameter. We therefore propose that the deceleration *a *can be evaluated starting from the confining mechanical pressure P exerted by the host tissue on the growing tumor. Assuming for simplicity a spherical shape for the tumor, where S and V are the surface and volume at the onset of invasion, respectively, we obtain:

a = PS/ρV = 3P/ρR     (3)

It follows that

N_f _= (PR/σ)^1/2 ^    (4)

The value N_f _= 1 separates the case of no branching (hence no tumor invasion) from that in which at least one branch develops (and invasion takes place). By defining the dimensionless *Invasion Parameter*, IP, as

**Figure 3:**
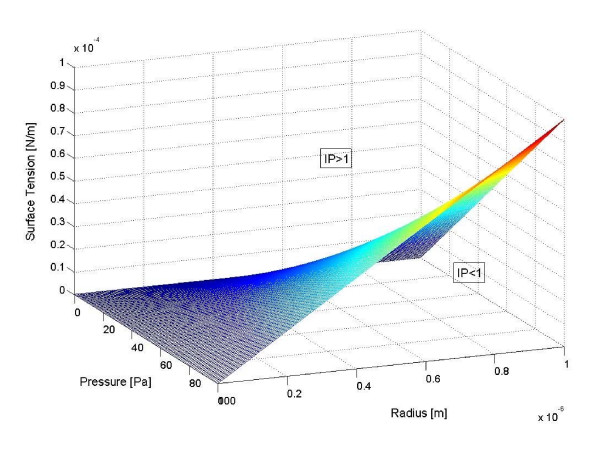
The surface IP = 1 according to Eqn. (5).

IP = PR/σ,     (5)

we deduce that, provided IP<1 (which implies large surface tension, small confining pressure and/or radius value) tumor invasion cannot occur, while for IP>1 invasive behaviour is expected (see Figure 3).

This index defines a critical value that predicts different potential outcomes of tumor growth instability regimen, i.e. self-similar growth versus invasive branching. The evaluation of the actual extent and/or rate of invasion may involve many other parameters which characterize the tumor growth processes as well as the microenvironment. In particular, provided the extent of invasion is related to the number of invasive branching, Eqn. (4) relates the 'invasion efficiency' to the square root of IP.

## Discussion

We appreciate the obvious differences between tumor biology and fluid dynamics. Yet, solely on the basis of the aforementioned perceived *analogy*, we argue here that the morphological instability that drives tumor invasion is controlled by a dimensionless parameter (IP) which is proportional to the confining pressure and tumor radius, yet inversely proportional to its surface tension. As a consequence, increasing levels of confinement at larger tumor radii should *promote *the onset of invasion, while larger values of adhesion-mediated surface tension can inhibit it. The former is in agreement with our previous, experimentally driven notion of a feedback between the key characteristics of proliferation and invasion [16] and the argument for a quantitative link between them [17]. The model's parameters can be measured and monitored, as well as modified with a treatment regimen. Intriguingly, the following ongoing experimental investigations seem already to confirm our conjectures:

### a) Tumor surface tension

Winters et al. [18] have investigated three different cells lines derived from malignant astrocytoma (U-87MG, LN-229 and U-118MG). Their work shows that (i) surface tension in the multicell aggregates they have used is independent of the compressive forces and that the spheres practically behave as a liquid and not as elastic aggregates; (ii) the measured aggregate surface tension is about 7 dyne/cm for U-87Mg, 10 for LN-229 and more than 16 for U-118MG, and (iii) there is indeed a significant *inverse *correlation between invasiveness and surface tension; (iv) finally, the anti-invasive therapeutic agent Dexamethasone increases the microscopic tumor's surface tension or cohesivity amongst cells in direct contact. For the cell lines studied above, surface tension is therefore a predictor for *in vitro *invasiveness and the authors suggest a threshold value for σ of about 10 dyne/cm. We add that other aspects of surface tension and intercellular adhesion have also been investigated, and shown to be relevant for non invasive tumor development (e.g. [19-21]).

### b) Microenvironmental pressure

Some papers have recently addressed the problem of the mechanical interaction of the matrix with the embedded tumor. For instance, Paszek et al. [22] claim that stiffer tissues are expected to promote malignant behaviour. Also, an experimental investigation by Georges and Janmey [23] shows that a basic NIH3T3 fibroblast embedded in a soft polyacrylamide gel develops with a roughly spherical shape (suggesting prevalence of cohesive forces), while in a stiff gel it exhibits finger-like features (consistent with the preponderance of adhesive forces). Another recent paper, by Kaufman et al. [24], investigates how glioblastoma spheroids grow in and invade 3D collagen I matrices, differing in collagen concentration and thus, in their average stiffness (the elastic modulus of the 0.5 mg/ml gel was 4 Pa, in the 1.0 mg/ml gel was 11 Pa and in the 1.5 mg/ml was about 100 Pa). Using *in vitro *microscopy techniques, the authors show that in the 0.5 mg/ml gel there are few invasive cells around the tumor. At larger concentrations (1.5–2.0 mg/ml gels) invasion occurs more quickly and the number of invasive cells increases. In conclusion, reducing the matrix stiffness seems to reduce the number of invasive cells and their invasion rate. It is noteworthy in this context that the relevance of local pressure in hindering the growth of non-invasive MTS has been studied by e.g. [25-27].

### c) Tumor radius

Tamaki et al. [13] investigated C6 astrocytoma spheroids with different diameters (i.e., 370, 535 and 855 μm on average) that were implanted in collagen type I gels. The authors showed that spheroid size indeed correlated with a larger total invasion distance and an increased rate of invasion. We note that, reflecting the complexity of the cancer system's expansion process properly [16,17], our concept relies on experimental conditions that allow for *both *cancer growth *and *invasion to occur. From Eqn. (5) it follows that, if invasion is restricted by the chosen experimental conditions and σ is assumed to remain constant, any increase in P beyond a certain threshold would result in limiting R. This is indeed confirmed by Helmlinger et al. [25], who reported that a solid stress of 45–120 mmHg inhibits the growth of multicellular tumor spheroids cultured in an agarose matrix (according to the authors, 'cells cannot digest or migrate through it').

## Conclusion

In summary, our model, while admittedly very simple, suggests – based on a striking fluid dynamics analogy – several clinical management strategies that, separately or in combination, should yield anti-invasive effects. They include (aside from the obvious initial attempt to *reduce the tumor size *through surgical techniques and accompanying non-surgical approaches (radio- and chemotherapy)):

### (1) Promoting tumor cell-tumor cell adhesion and thus increasing the tumor surface tension σ

Interestingly, experiments on prostate cancer cells have already shown that stable transfection of E-cadherin (the prototype cell-cell adhesion molecule that is increasingly lost with tumor progression) results in cellular cohesiveness and a decrease in invasiveness, in part due to a down-regulation of matrix metalloproteinase (MMP) activity [28]. Such a functional relationship (and thus our argument to capitalize on it for therapeutic purposes) is further supported by results from squamous cell carcinoma cells that had been genetically engineered to stably express a dominant-negative E-cadherin fusion protein [29]. The authors reported that, in three-dimensional environments, E-cadherin deficiency indeed led to a loss of intercellular adhesion and triggered tumor cell invasion by MMP-2 and MMP-9 driven matrix degradation.

### (2) Reducing the confining mechanical pressure exerted on the tumor

This refers to pharmacological strategies that range from applying perioperatively corticosteroids, as it is standard for treating malignant brain tumors [30], to preventing pressure-stimulated cell adhesion, i.e. mechanotransduction by targeting the cytoskeleton's actin polymerization [31,32].

Taken together, our model is not only supported by a variety of experimental findings, but it offers already an explanation for the anti-invasive and anti-metastatic effects seen in the aforementioned experimental studies and clinical regimen, respectively. As such, this model has the potential to further our understanding of the dynamical relationship between a tumor and its microenevironment, and, in its future iterations, may even hold promise for assessing the potential impact of combinatory treatment approaches.

## Competing interests

The author(s) declare that they have no competing interests.

## Authors' contributions

All authors contributed equally to this work. All have read and approved the final manuscript.
